# Metformin Treatment Attenuates Brain Inflammation and Rescues PACAP/VIP Neuropeptide Alterations in Mice Fed a High-Fat Diet

**DOI:** 10.3390/ijms222413660

**Published:** 2021-12-20

**Authors:** Mawj Mandwie, Jocelyn Karunia, Aram Niaz, Kevin A. Keay, Giuseppe Musumeci, Claire Rennie, Kristine McGrath, Ghaith Al-Badri, Alessandro Castorina

**Affiliations:** 1 Laboratory of Cellular and Molecular Neuroscience (LCMN), School of Life Sciences, Faculty of Science, University of Technology Sydney, Sydney, NSW 2007, Australia; mmandwie@cmri.org.au (M.M.); jocelyn.karunia@uts.edu.au (J.K.); aram.niaz@health.nsw.gov.au (A.N.); g.al-badri@unsw.edu.au (G.A.-B.); 2 Laboratory of Neural Structure and Function, School of Medical Science (Neuroscience), University of Sydney, Sydney, NSW 2006, Australia; kevin.keay@sydney.edu.au; 3 Department of Biomedical and Biotechnological Sciences, Anatomy, Histology and Movement Sciences Section, School of Medicine, University of Catania, 95125 Catania, Italy; g.musumeci@unict.it; 4 School of Life Sciences, Faculty of Science, University of Technology Sydney, Sydney, NSW 2007, Australia; claire.rennie@uts.edu.au (C.R.); kristine.mcgrath@uts.edu.au (K.M.)

**Keywords:** high-fat diet, PACAP, VIP, prefrontal cortex, hippocampus, hypothalamus, amygdala, metformin, PI3K/AKT, neuroinflammation

## Abstract

High-fat diet (HFD)-induced comorbid cognitive and behavioural impairments are thought to be the result of persistent low-grade neuroinflammation. Metformin, a first-line medication for the treatment of type-2 diabetes, seems to ameliorate these comorbidities, but the underlying mechanism(s) are not clear. Pituitary adenylate cyclase-activating peptide (PACAP) and vasoactive intestinal peptide (VIP) are neuroprotective peptides endowed with anti-inflammatory properties. Alterations to the PACAP/VIP system could be pivotal during the development of HFD-induced neuroinflammation. To unveil the pathogenic mechanisms underlying HFD-induced neuroinflammation and assess metformin’s therapeutic activities, (1) we determined if HFD-induced proinflammatory activity was present in vulnerable brain regions associated with the development of comorbid behaviors, (2) investigated if the PACAP/VIP system is altered by HFD, and (3) assessed if metformin rescues such diet-induced neurochemical alterations. C57BL/6J male mice were divided into two groups to receive either standard chow (SC) or HFD for 16 weeks. A further HFD group received metformin (HFD + M) (300 mg/kg BW daily for 5 weeks) via oral gavage. Body weight, fasting glucose, and insulin levels were measured. After 16 weeks, the proinflammatory profile, glial activation markers, and changes within the PI3K/AKT intracellular pathway and the PACAP/VIP system were evaluated by real-time qPCR and/or Western blot in the hypothalamus, hippocampus, prefrontal cortex, and amygdala. Our data showed that HFD causes widespread low-grade neuroinflammation and gliosis, with regional-specific differences across brain regions. HFD also diminished phospho-AKT^(Ser473)^ expression and caused significant disruptions to the PACAP/VIP system. Treatment with metformin attenuated these neuroinflammatory signatures and reversed PI3K/AKT and PACAP/VIP alterations caused by HFD. Altogether, our findings demonstrate that metformin treatment rescues HFD-induced neuroinflammation in vulnerable brain regions, most likely by a mechanism involving the reinstatement of PACAP/VIP system homeostasis. Data also suggests that the PI3K/AKT pathway, at least in part, mediates some of metformin’s beneficial effects.

## 1. Introduction

Obesity has emerged as a major health issue both in industrialized and developing countries [1]. In the absence of genetic predisposition, obesity is gradually achieved when the daily ingested calories surpass the caloric burn, thus its development is largely attributed to overeating, inadequate diet, insufficient physical activity, or their combination [2]. A high-fat diet (HFD) induces several metabolic changes in the periphery, which promote a generalized inflammatory state in tissues driven by the ingress and activation of immune cells [3]. In the central nervous system (CNS), a similar inflammatory state has been observed primarily in the hypothalamus, a critical brain region regulating energy homeostasis and food intake [4,5,6]. The hypothalamus has been the primary focus of studies evaluating the inflammatory effects of HFD, with few studies evaluating impacts on extra-hypothalamic regions.

Several studies have associated HFD with impairments in executive function and memory [7,8,9,10], as well as an increased incidence of mood disorders, such as anxiety [11,12] and depression [13,14]. Most recently, HFD has been shown to exacerbate the risk of neurodegenerative diseases [15,16,17]. HFD-animal models exhibit signs of learning and memory impairment [18,19,20,21], as well as depressive and anxiety-like behaviors [22]. The severity of behavioral impairments observed in these animal models has prompted a number of laboratories to explore whether the disruptions caused by HFD could exceed the “mild hypothalamic inflammation” described so far [23]. 

HFD causes persistent low-grade hypothalamic inflammation, characterized by increased cytokine production, neuronal stress, and insulin/leptin resistance [4,24]. The functional changes in the hypothalamus occur due to the destruction of tight junctions in the micro-vascular compartment of the blood brain barrier (BBB) and increased homing and migration of peripheral inflammatory cells, resulting in the release of proinflammatory cytokines [25,26]. Notwithstanding the importance of the hypothalamus in HFD-induced neurocognitive issues, several other brain regions including the prefrontal cortex (PFC), hippocampus, and amygdala have been identified as vulnerable to the detrimental effects of HFD. Preclinical studies have suggested that HFD reduces synaptic plasticity in the PFC [27] and hippocampus [28,29], which results in impairments in both learning and memory [30]. Moreover, there is evidence that HFD reduces structural integrity of the PFC and the amygdala [31,32], two brain regions critical for the control of appetite and a range of other motivated behaviors [33,34]. Despite these findings, therapeutic interventions targeting HFD-induced neuroinflammation and its associated cognitive decline are lacking.

One drug with clear beneficial effects, acting directly on the CNS, is metformin (1,1-dimethylbiguanide). Metformin is an inexpensive, safe, and well-tolerated anti-diabetic drug currently prescribed as a first-line therapy to treat type-2 diabetes (T2D) [35]. Studies have revealed that metformin promotes neurogenesis and enhances spatial memory in C57/129J mice [36]. Additionally, acute metformin preconditioning is neuroprotective against cerebral ischemia [37]. More recently, metformin has been found to possess anti-inflammatory activities in the CNS [38,39]. These activities appear to be mediated by the induction of the adenosine monophosphate (AMP)-activated protein kinase (AMPK) [38]. In contrast, there are a several studies that have suggested that suppression of the critical phosphoinositide 3-kinase (PI3K)/protein kinase B (AKT) pathway might be implicated with the detrimental effects of HFD in the brain, given its multiple beneficial roles in the CNS, including neuroprotective activities [40], energy metabolism, and synaptic plasticity [41]. Intriguingly, AMPK, the main target of metformin, is also a potent inducer of the PI3K/AKT cascade. Yet, whether HFD can actually suppress the PI3K/AKT cascade and whether metformin can reverse HFD effects via its activity on AMPK have not been demonstrated. Additionally, the effect of HFD on other known upstream regulators of the beneficial PI3K/AKT pathway in the CNS still warrants investigations. 

The pituitary adenylate cyclase-activating polypeptide/vasoactive intestinal peptide (PACAP/VIP) system is well recognized for its potent neuroprotective and anti-inflammatory actions in vitro and in vivo [42,43,44,45,46]. PACAP and VIP are two neuropeptides that exert their actions through their receptors PAC1, VPAC1, and VPAC2. The PACAP/VIP system plays a critical role in CNS protection [44,47,48,49]. These neuropeptides are involved in the regulation of feeding behavior and have anti-obesogenic effects [50,51], and they also promote the activation of the PI3K/AKT cascade [52,53]. In neural tissues, PACAP-38 is the dominant form of the peptide [54,55]. It is involved in numerous biological processes, including vasodilation, endocrine regulation, and hippocampal function [56,57,58,59]. Similarly, VIP is a 28-amino acid peptide isolated initially from porcine intestines, where it acts as a potent vasodilator [60,61]. VIP functions as a neuroendocrine hormone, a putative neurotransmitter, and a cytokine. The presence of pathway-specific VIP and VIP binding sites in the CNS highlights an important role in CNS function [62,63]. Similar to PACAP, VIP is involved in promoting neuronal survival [64]; it also elicits robust anti-inflammatory [65] and immune-suppressive activities [66]. Although these two peptidergic systems have been the subject of intense functional analyses, there are no data yet concerning the effects of HFD on the regulation of this critical endogenous neuroprotective system. 

To date, there is little known about the brain changes underlying HFD-associated cognitive comorbidities. To begin to address this knowledge gap, we tested the hypothesis that exposure to HFD triggers chronic low-grade neuroinflammation in the hypothalamus and other brain regions critical for cognitive functioning and known to be vulnerable to neuroinflammation. These regions were further explored for the co-expression of disruptions to the PACAP/VIP system and the PI3K/AKT intracellular pathway with regions of HFD-evoked neuroinflammation. A series of studies evaluated the following hypotheses:

(1) HFD mice show signatures of neuroinflammation in the hypothalamus and other vulnerable extra-hypothalamic CNS sites;
(2) The PACAP/VIP neuropeptide system is dysregulated by HFD in brain regions showing HFD-triggered neuroinflammations; and
(3) The antidiabetic drug metformin ameliorates neuroinflammation by rescuing HFD-induced dysregulation of the PACAP/VIP system and/or the PI3K/AKT pathways.

## 2. Results

### 2.1. Metformin Treatment Attenuated HFD-Induced Glucose Tolerance, Insulin Resistance, and Weight Gain 

**Figure 1:**
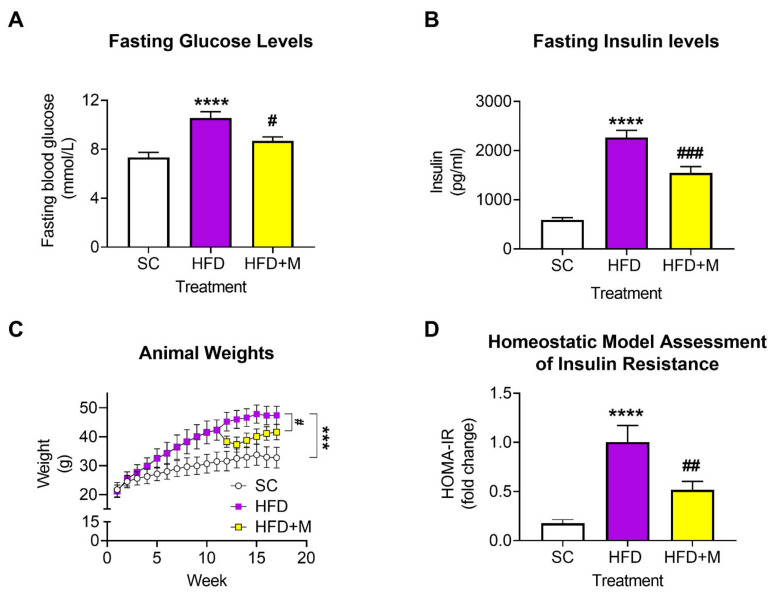
Fasting blood glucose and insulin levels, body weight, and insulin tolerance in mice fed with HFD and/or treated with metformin. (**A**,**B**) Fasting glucose and insulin levels were measured in animals fed with standard chow, HFD, or HFD plus metformin (300 mg/kg/BW, oral gavage) as per the manufacturer’s protocols. Tests were conducted after 5 h of fasting at the end of week 17. (**C**) Animal weights were monitored on a weekly basis and measurements were recorded. (**D**) Homeostatic model assessment for insulin resistance index (HOMA-IR) was calculated using the following equation, HOMA-IR = fasting glucose (mg/dL) × fasting insulin (μU/mL)/405. *** *p* < 0.001 or **** *p* < 0.0001 vs. SC; # *p* < 0.05, ## *p* < 0.01 or ### *p* < 0.001 vs. HFD, as determined by one-way ANOVA followed by Tukey’s post-hoc test. SC: Standard Chow, HFD: High Fat Diet, HFD + M: High Fat Diet + metformin.

To assess whether mice developed hyperinsulinemia in response to HFD, and to test whether metformin treatment reversed these effects, fasting glucose and insulin levels were measured. In addition, Homeostatic Model Assessment of Insulin Resistance (HOMA-IR) was also calculated. The bodyweights of the mice were measured each week for the duration of the study to identify any changes in response to either dietary regimes (SC or HFD) or drug treatments (HFD + M). Comparative analyses showed that HFD-treated mice had significantly increased blood glucose levels, which were reversed by metformin treatment (Figure 1A, **** *p* < 0.0001 vs. SC and # *p* < 0.05 vs. HFD, respectively). The HFD regime also increased fasting insulin levels (**** *p* < 0.001 vs. SC), which were significantly lowered by metformin treatment (Figure 1B, ### *p* < 0.001 vs. HFD). 

As expected, mice subjected to the HFD regime displayed a significant weight gain when compared with SC-fed mice (Figure 1C, F_(2,48)_ = 8.410, *** *p* < 0.001 vs. SC). Commencement of metformin treatment at week 11 in the HFD group significantly reduced HFD-induced weight gain (# *p* < 0.05 vs. HFD). HOMA-IR results demonstrated a significant increase in insulin resistance following HFD (Figure 1D, **** *p* < 0.0001 vs. SC), whereas in metformin-treated animals (HFD + M), insulin resistance significantly decreased (Figure 1D, ## *p* < 0.01 vs. HFD).

### 2.2. Metformin Treatment Rescues HFD-Induced Neuroinflammation and Glial Cell Activation in a Region-Specific Manner

To assess if HFD triggered different neuroinflammatory profiles across brain regions critical for motivated and cognitive behaviors and to determine whether metformin treatment reversed these effects, we performed real-time qPCR analyses and Western blots on four ROI: the hypothalamus, hippocampus, prefrontal cortex, and amygdala. Three groups were used for gene and protein expression analyses: SC (*n* = 4–8), HFD (*n* = 6–8), and HFD + M (*n* = 6–8). All real-time qPCR analyses were performed on tissue blocks microdissected from the left side of the brain. Gene expression studies were conducted to investigate several inflammatory markers (IL-1α, IL-1β, IL-6, TNF, Mcp1, IFN-γ, GFAP, Iba1, CD68). For Western blots, we interrogated the same brain regions as in qPCR studies but using blocks obtained from the right side of the brain. We probed the glial-specific markers Iba1, GFAP, and iNOS (*n* = 4 mice per each group). GAPDH was used as a loading control.

**Figure 2:**
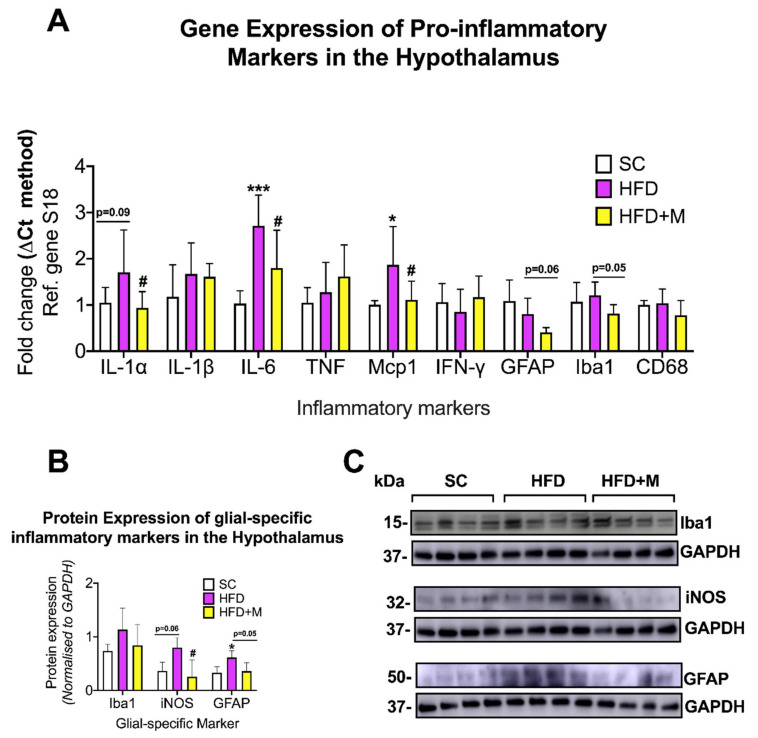
Gene and protein expression analysis of cytokines and glial-specific inflammatory markers in the hypothalamus of C57BL/6 mice in response to HFD or metformin administration. Mice were grouped as SC, HFD, and HFD + M. Hypothalami were microdissected from the left and right sides of the brain from all treatment groups and were used for RNA and protein extraction with subsequent quantitative qPCR and Western blotting, respectively. (**A**) Differential gene expression levels of proinflammatory mediators and glial-specific inflammatory markers. (**B**) Protein expression levels of glial-specific inflammatory marker following normalization to the loading control, GAPDH. (**C**) Immunoblot bands for each marker. mRNA levels were quantified using the ΔCt method and normalized to the reference gene S18 (housekeeping gene). Data were presented as mean ± S.E.M. * *p* < 0.05 or *** *p* < 0.001 vs. SC group; # *p* < 0.05 vs. HFD group as determined by one-Way ANOVA followed by Tukey post-hoc test. *n* = 6–8 per group. Quantification of immunoblot bands was performed using the NIH ImageJ software. Data were given as mean ± S.E.M. * *p* < 0.05 vs. SC; # *p* < 0.05 vs. HFD group as determined by One-Way ANOVA followed by Tukey post-hoc test. *n* = 4 per group. *Mcp1*: Monocyte Chemoattractant Protein-1, *IL-1α*: Interleukin 1alpha, *IFN-γ*: Interferon gamma, *IL-1β*: Interleukin 1beta, *GFAP*: Glial Fibrillary Acidic Protein, *IL-6*: Interleukin 6, *Iba1*: Ionized calcium-Binding Adapter molecule 1, *TNF*: Tumor Necrosis Factor, *CD68*: Cluster of Differentiation 68, *S18*: 40S ribosomal protein S18, SC: Standard Chow, HFD: High-Fat Diet, HFD + M: High-Fat Diet + metformin, Ref: Reference. iNOS: inducible Nitric Oxide Synthase, Iba1: Ionized Calcium-Binding Adapter molecule 1, GFAP: Glial Fibrillary Acidic Protein, GAPDH: Glyceraldehyde3-phosphate dehydrogenase, kDa: Kilodalton.

Hypothalamus: Both IL-6 and Mcp1 mRNA levels were significantly upregulated in the HFD group (Figure 2A, F_(2,17)_ = 9.978, *** *p* < 0.001, F_(2,19)_ = 5.299, * *p* < 0.05 vs. SC, respectively). A similar although not statistically significant increase was observed for IL-1α (Figure 2A, F_(2,21)_ = 3.898, *p* = 0.094 vs. SC). GFAP and Iba1 mRNA levels showed no statistically significant changes in the HFD group (*p* > 0.05 vs. SC). Metformin treatment reversed the HFD-driven increase in IL-1α, IL-6, and Mcp1 mRNAs (Figure 2A, # *p* < 0.05 vs. HFD). Although not statistically significant, GFAP and Iba1 transcripts were also clearly diminished by metformin treatment (Figure 2A, F_(2,21)_ = 8.148, *p* = 0.068, F_(2,21)_ = 3.237, *p* = 0.0516 vs. HFD, respectively). IL-1β, TNF, IFN-γ, and CD68 mRNAs were unchanged across the groups (*p* > 0.05). 

GFAP protein expression was significantly upregulated in the HFD group (Figure 2B,C, F_(2,19)_ = 5.606, * *p* < 0.05 vs. SC), whereas both Iba1 and iNOS protein levels were only marginally increased (Figure 2B,C, F_(2,19)_ = 6.253, *p* > 0.05 and *p* = 0.0606, respectively). Metformin treatment decreased both GFAP and iNOS protein expression levels when compared with HFD (Figure 2B,C, # *p* < 0.05 and *p* = 0.0554, respectively). Iba1 protein expression was also reversed by drug treatment but not in a statistically significant manner (*p* > 0.05).

**Figure 3:**
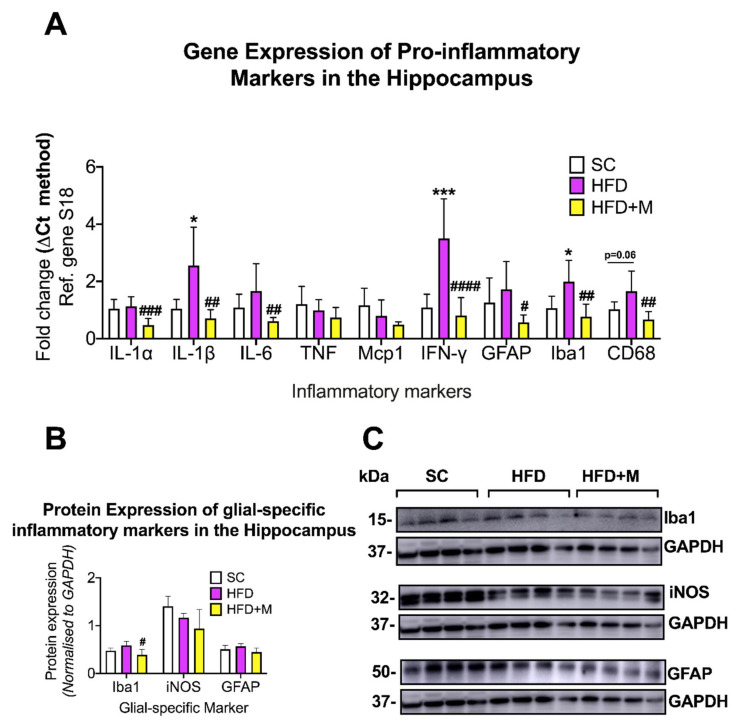
Gene and protein expression analysis of cytokines and glial-specific inflammatory markers in the hippocampus of C57BL/6 mice in response to HFD or metformin administration. Mice were grouped as SC, HFD, and HFD + M. Hippocampi were microdissected from the left and right sides of the brain from all treatment groups and were used for RNA and protein extraction with subsequent quantitative qPCR and Western blotting, respectively. (**A**) Differential gene expression levels of proinflammatory mediators and glial-specific inflammatory markers. (**B**) Protein expression levels of glial-specific inflammatory marker following normalization to the loading control, GAPDH. (**C**) Immunoblot bands for each marker. mRNA levels were quantified using the ΔCt method and normalized to the reference gene S18 (housekeeping gene). Data were presented as mean ± S.E.M. * *p* < 0.05 or *** *p* < 0.001 vs. SC group; # *p* < 0.05, ## *p* < 0.01, ### *p* < 0.001, or #### *p* < 0.0001 vs. HFD group as determined by one-way ANOVA followed by Tukey post-hoc test. *n* = 6–8 per group. Quantification of immunoblot bands was performed using the NIH ImageJ software. Data were given as mean ± S.E.M. # *p* < 0.05 vs. HFD group as determined by one-way ANOVA followed by Tukey post-hoc test. *n* = 4 per group.

Hippocampus: HFD significantly increased IL-1β (F_(2,18)_ = 9.437, * *p* < 0.05), IFN-γ (F_(2,19)_ = 19.91, *** *p* < 0.001) and Iba1 mRNAs (F_(2,19)_ = 9.942, * *p* < 0.05) when compared with SC (Figure 3A). CD68 mRNA was also upregulated by the HFD (Figure 3A), F_(2,19)_ = 8.666, *p* = 0.0629 vs. SC). Metformin treatment diminished IL-1α (F_(2,19)_ = 11.62, #### *p* < 0.001 vs. HFD), IL-1β (F_(2,19)_=5.77, ## *p* < 0.01), IL-6 (F_(2,19)_ = 5.68, ## *p* < 0.01), IFN-γ (#### *p* < 0.0001), Iba1 (## *p* < 0.01), and CD68 (## *p* < 0.01) (Figure 3A). 

Iba1 protein expression was slightly upregulated by the HFD and metformin treatment returned protein expression back to SC levels, an effect that was statistically significant (Figure 3B,C, F_(2,19)_ = 5.128, # *p* < 0.05 vs. HFD). 

**Figure 4:**
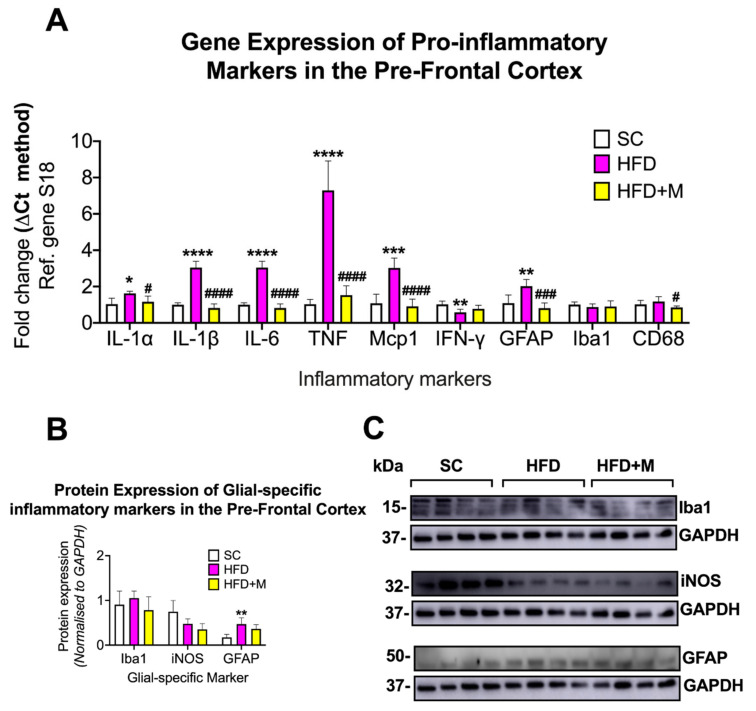
Gene and protein expression analysis of cytokines and glial-specific inflammatory markers in the prefrontal cortex of C57BL/6 mice in response to HFD or metformin administration. Mice were grouped as SC, HFD, and HFD + M. Pre-frontal cortices were microdissected from the left and right sides of the brain from all treatment groups and were used for RNA and protein extraction with subsequent quantitative qPCR and Western blotting, respectively. (**A**) Differential gene expression levels of proinflammatory mediators and glial-specific inflammatory markers. (**B**) Protein expression levels of glial-specific inflammatory marker following normalization to the loading control, GAPDH. (**C**) Immunoblot bands for each marker. mRNA levels were quantified using the ΔCt method and normalized to the reference gene S18 (housekeeping gene). Data were presented as mean ± S.E.M. * *p* < 0.05, ** *p* < 0.01, *** *p* < 0.001, or **** *p* < 0.0001 vs. SC; # *p* < 0.05, ### *p* < 0.001 or #### *p* < 0.0001 vs. HFD group as determined by one-way ANOVA followed by Tukey post-hoc test. *n* = 4–8 per group. Quantification of immunoblot bands was performed using the NIH ImageJ software. Data were given as mean ± S.E.M. ** *p* < 0.01 vs. SC group as determined by one-way ANOVA followed by Tukey post-hoc test. *n* = 4 per group.

Prefrontal Cortex (PFC): In the PFC, HFD increased the expression of several proinflammatory marker genes. Specifically, the expression of IL-1α (F_(2,15)_ = 5.319, * *p* < 0.05), IL-1β (F_(2,13)_ = 63.9, **** *p* < 0.0001), IL-6 (F_(2,13)_ = 120, **** *p* < 0.0001), TNF (F _(2,13)_ = 68.63, **** *p* < 0.0001), Mcp1 (F_(2,11)_ = 27.58, *** *p* < 0.001), and GFAP mRNA’s (F_(2,15) =_ 14.8, ** *p* < 0.01) were all significantly increased compared with the SC group (Figure 4A). In contrast, IFN-γ was significantly downregulated in the HFD group (Figure 4A, F_(2,15)_ = 6.917, ** *p* < 0.01). Administration of metformin to HFD animals resulted in a significant decrease of IL-1α (# *p* < 0.05), IL-1β (#### *p* < 0.0001), IL-6 (#### *p* < 0.0001), TNF (#### *p* < 0.0001), Mcp1 (#### *p* < 0.0001), GFAP (### *p* < 0.001), and CD68 mRNA expression (F_(2,15)_ = 4.512, # *p* < 0.05) (Figure 4A). 

GFAP protein expression was significantly increased by HFD (F_(2,9)_ = 8.098, ** *p* < 0.01 vs. SC) but only modestly reduced by metformin administration (Figure 4B,C, *p* > 0.05 vs. HFD). 

**Figure 5:**
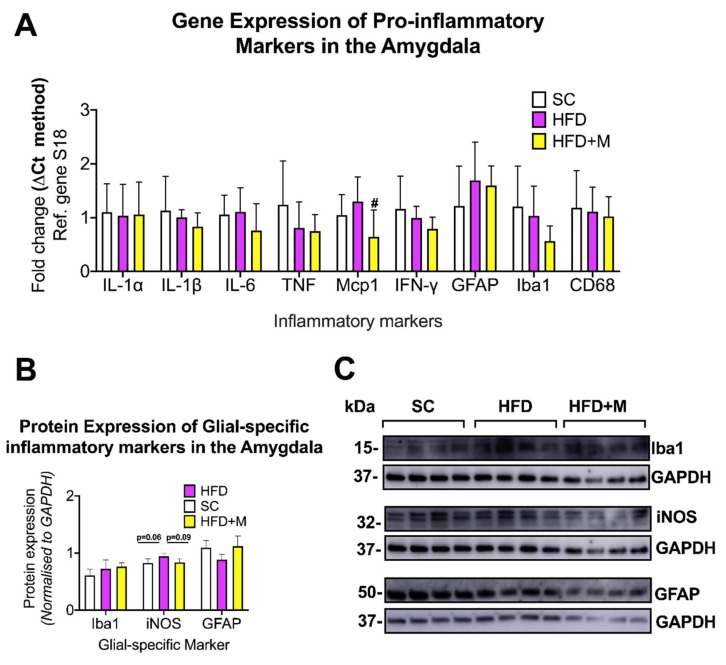
Gene and protein expression analysis of cytokines and glial-specific inflammatory markers in the amygdala of C57BL/6 mice in response to HFD or metformin administration. Mice were grouped as SC, HFD, and HFD + M. Amygdala were microdissected from the left and right sides of the brain from all treatment groups and were used for RNA and protein extraction with subsequent quantitative qPCR and Western blotting, respectively. (**A**) Differential gene expression levels of proinflammatory mediators and glial-specific inflammatory markers. (**B**) Protein expression levels of glial-specific inflammatory marker following normalization to the loading control, GAPDH. (**C**) Immunoblot bands for each marker. mRNA levels were quantified using the ΔCt method and normalized to the reference gene S18 (housekeeping gene). Data were presented as mean ± S.E.M. # *p* < 0.05 vs. HFD group as determined by one-way ANOVA followed by Tukey post-hoc test. *n* = 6–8 per group. Quantification of immunoblot bands was performed using the NIH ImageJ software. Data were given as mean ± S.E.M. P values were determined by one-way ANOVA followed by Tukey post-hoc test. *n* = 4 per group.

Amygdala: HFD did not cause any significant changes to IL-1α, IL-1β, IL-6, TNF, Mcp1, IFN-y, GFAP, Iba1, and CD68 gene expression (Figure 5A, *p* > 0.05). Metformin treatment had no detectable effects on the inflammatory profile of this brain region, with the exception of Mcp1 mRNAs, whose levels were reduced (Figure 5A, F_(2,18)_ = 3.922, # *p* < 0.05 vs. HFD). 

Western blots showed no significant changes in GFAP and Iba1 protein expression across all groups, with a slight increase only in iNOS protein expression (Figure 5B,C, F_(2,9)_ = 4.123, *p* = 0.06 vs. SC). Metformin slightly reduced iNOS protein levels when compared with HFD animals (Figure 5B,C, *p* = 0.09 vs. HFD).

### 2.3. Diet and Metformin Treatment Have No Effect on the mRNA Expression of the Anti-Inflammatory Cytokine IL-10 

To determine whether the anti-inflammatory cytokine IL-10 played a role in the pro- and anti-inflammatory activities of HFD and metformin treatment, respectively, we analyzed IL-10 mRNA expression by real-time qPCR. Our analyses did not reveal any significant changes in IL-10 mRNA levels in the four brain regions (Figure S1A–D, *p* > 0.05 vs. SC or vs. HFD). However, we did detect a minor, although not statistically significant, reduction of IL-10 transcripts in the hippocampus of animals that were treated with metformin (Figure S1B, *p* = 0.06 vs. HFD). 

### 2.4. Metformin Treatment Triggers the Increase of Phospho-Akt^(Ser473)^ Protein Levels in the Brain of Mice Fed with a HFD

To investigate the effects of HFD and metformin treatment on the activation of the PI3K/AKT pathway, we measured the levels of p-AKT^(Ser473)^ and total AKT by Western blot in all the four ROI under investigation. 

**Figure 6:**
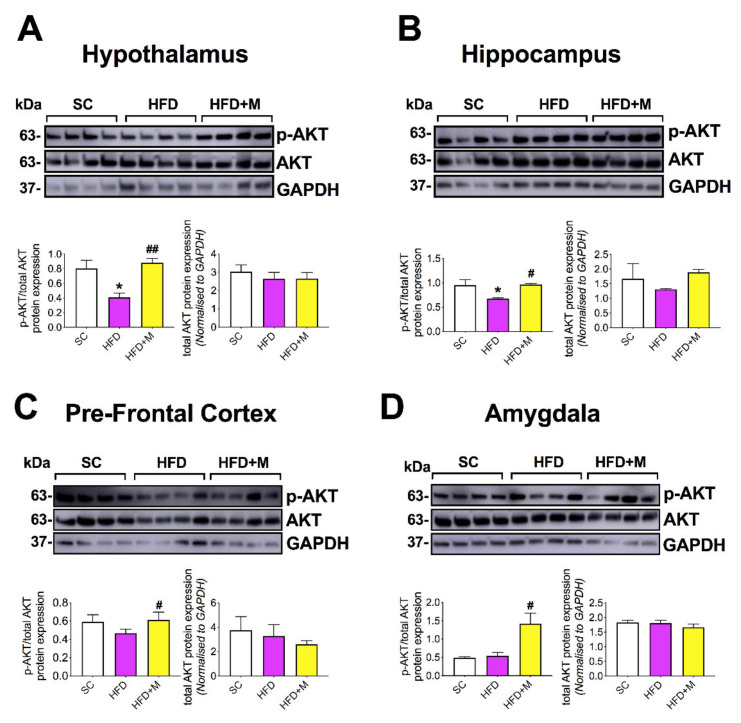
Effects of HFD and metformin administration on the phosphorylation of AKT at the Ser473 residue in the brain of C57BL/6 mice. Mice were grouped as SC, HFD, and HFD + M. Hypothalami, hippocampi, pre-frontal cortices, and amygdala were microdissected from the right side of the brain from all treatment groups and were used for protein extraction and subsequent Western blotting. p-AKT protein expression in (**A**) the hypothalamus, (**B**) hippocampus, (**C**) pre-frontal cortex, and (**D**) amygdala were represented in immunoblot bands for p-AKT, biological control total AKT, and loading control GAPDH. Bar graphs were also blotted for p-AKT following normalization to the biological control, total AKT (left), and a bar graph depicting total AKT protein normalized to the loading control, GAPDH (right). Quantification of blots was performed using the NIH ImageJ software. Data were given as mean ± S.E.M. * *p* < 0.05 vs. SC; # *p* < 0.05 vs. HFD group; ## *p* < 0.01 vs. HFD group as determined by one-way ANOVA followed by Tukey post-hoc test. *n* = 4 per group.

HFD significantly downregulated p-AKT activity in the hypothalamus (Figure 6A, F_(2,9)_ = 9.656 * *p* < 0.05 vs. SC), which was completely rescued by metformin (Figure 6A, ## *p* < 0.01 vs. HFD). In the hippocampus, p-AKT activity was also significantly reduced in the HFD group (Figure 6B, F_(2,9)_ = 5.565, * *p* < 0.05 vs. SC), and identical to the hypothalamus, metformin treatment rescued the hippocampal reduction of p-AKT activity caused by HFD (Figure 6B, # *p* < 0.05 vs. HFD). In the PFC, p-AKT activity was only slightly reduced in the HFD group (Figure 6C, *p* > 0.05); however, metformin significantly increased p-AKT activation (Figure 6C, # *p* < 0.05 vs. HFD). This activation was at levels similar to those seen in the SC group. In the amygdala, HFD had no effect on p-AKT activity; however, mice treated with metformin displayed a significant upregulation of p-AKT when compared with HFD animals (Figure 6D, F_(2,9)_ = 9.078, # *p* < 0.05). 

### 2.5. Metformin Administration Rescues HFD-Induced Dysregulations of the PACAP/VIP Neuropeptide System 

To determine if the neuroprotective PACAP/VIP system was dysregulated by HFD and to evaluate whether metformin administration could reverse these changes, we measured the mRNA and protein expression of *Adcyap1* (PACAP), *Vip* (VIP), *Adcyap1r1* (PAC1), *Vipr1* (VPAC1), and *Vipr2* (VPAC2) in the four ROIs using both real-time qPCR and Western blots. 

**Figure 7:**
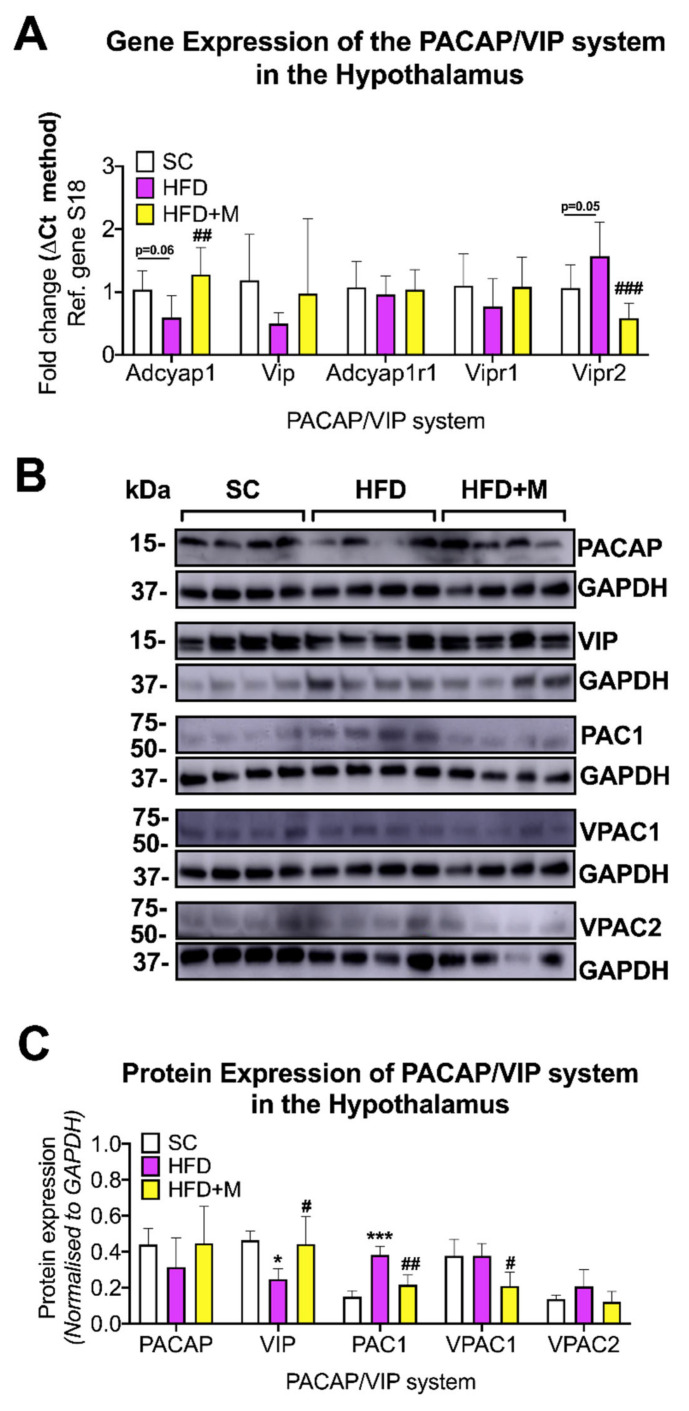
Gene and protein expression analysis of the PACAP/VIP system in the hypothalamus of C57BL/6 mice in response to HFD or metformin administration. Mice were grouped as SC, HFD, and HFD + M. Hypothalami were microdissected from the left and right sides of the brain from all treatment groups and were used for RNA and protein extraction with subsequent quantitative qPCR and Western blotting, respectively. (**A**) Differential gene expression levels of the PACAP/VIP system, (**B**) Immunoblot bands for PACAP/VIP system members were represented. (**C**) Protein expression levels of the PACAP/VIP system following normalization to the loading control, GAPDH. mRNA levels were quantified using the ΔCt method and normalized to the reference gene S18 (housekeeping gene). Data were given as mean ± S.E.M. ## *p* < 0.01 or ### *p* < 0.001 vs. HFD group as determined by One-Way ANOVA followed by Tukey post-hoc test. *n* = 6–8 per group. Quantification of immunoblot bands was performed using the NIH ImageJ software. Data were given as mean ± S.E.M. * *p* < 0.05 or *** *p* < 0.001 vs. SC group; # *p* < 0.05 or ## *p* < 0.01 vs. HFD group as determined by one-way ANOVA followed by Tukey post-hoc test. *n* = 4 per group. SC: Standard Chow, HFD: High-Fat Diet, HFD + M: High-Fat Diet + metformin, GAPDH: Glyceraldehyde3-phosphate dehydrogenase, PACAP: Pituitary Adenylate Cyclase-Activating Peptide, VIP: Vasoactive Intestinal Polypeptide, Adcyap1: Adenylate cyclase activating polypeptide 1, kDa: Kilodalton, S18: 40S ribosomal protein S18, Ref: Reference.

Hypothalamus: HFD caused a slight decrease in *Adcyap1* gene expression (Figure 7A, F_(2,21)_ = 7.194, *p* = 0.0606), which was reversed by metformin treatment (Figure 7A, ## *p* < 0.01 vs. HFD). *Vipr2* gene expression was increased by HFD (Figure 7A, F_(2,21)_ = 12.06 *p* = 0.05) and decreased to SC levels by metformin treatment (Figure 7A, ### *p* < 0.001 vs. HFD). 

Despite similar trends in the mRNA data, the changes in PACAP protein expression were not statistically significant among groups (Figure 7B,C, *p* > 0.05). In contrast, VIP protein expression was significantly decreased by HFD (F_(2,9)_ = 5.932, * *p* < 0.05 vs. SC) and was reliably increased after metformin treatment (# *p* < 0.05 vs. HFD) (Figure 7B,C). We also found that PAC1 protein expression was significantly increased in the HFD group (Figure 7B,C, F_(2,9)_ = 26.89, *** *p* < 0.001), but it returned to SC levels in animals that received metformin (## *p* < 0.01 vs. HFD). VPAC1 expression was not affected in the HFD group, and its expression was significantly reduced in mice receiving metformin (Figure 7B,C, F_(2,9)_ = 5.85, # *p* < 0.05). 

**Figure 8:**
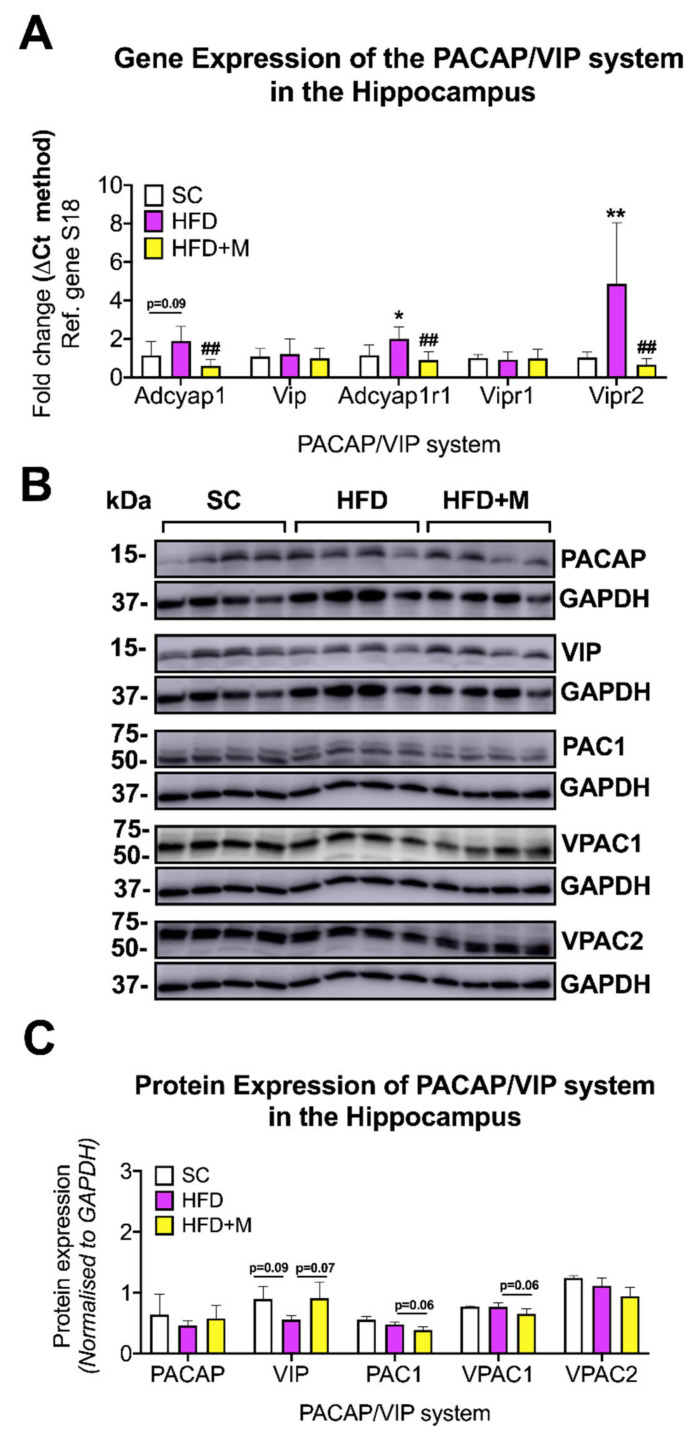
Gene and protein expression analysis of the PACAP/VIP system in the hippocampus of C57BL/6 mice in response to HFD or metformin administration. Mice were grouped as SC, HFD, and HFD + M. Hippocampi were microdissected from the left and right sides of the brain from all treatment groups and were used for RNA and protein extraction with subsequent quantitative qPCR and Western blotting, respectively. (**A**) Differential gene expression levels of the PACAP/VIP system, (**B**) Immunoblot bands for PACAP/VIP system members were represented and (**C**) protein expression levels of the PACAP/VIP system following normalization to the loading control, GAPDH. mRNA levels were quantified using the ΔCt method and normalized to the reference gene S18 (housekeeping gene). Data were given as mean ± S.E.M. * *p* < 0.05 or ** *p* < 0.01 vs. SC group, ## *p* < 0.01 vs. HFD group as determined by one-way ANOVA followed by Tukey post-hoc test. *n* = 6–8 per group. Quantification of immunoblot bands was performed using the NIH ImageJ software. Data were given as mean ± S.E.M. Data were given as mean ± S.E.M. *p* < 0.05 were regarded statistically significant as determined by one-way ANOVA followed by Tukey post-hoc test. *n* = 4 per group.

Hippocampus: Hippocampal expression of *Adcyap1r1* and *Vipr2* mRNA was each robustly increased in response to HFD (Figure 8A, F_(2,20)_ = 9.246 and F_(2,15)_ = 9.547, * *p* < 0.05 and ** *p* < 0.01 vs. SC, respectively), whereas *Adcyap1* gene levels were only marginally elevated (F_(2,19)_ = 8.74, *p* = 0.09). Interestingly, metformin reversed all the HFD-driven increases in gene expression, significantly reducing both *Adcyap1*, *Adcyap1r1*, and *Vipr2* gene expression to control levels (Figure 8A, ## *p* < 0.01 vs. HFD). Western blot analyses of hippocampal tissues were not absolutely consistent with mRNA expression changes. No significant changes were observed in PACAP protein expression across the treatment groups (Figure 8B,C, *p* > 0.05). VIP was slightly decreased by the HFD and increased in animals that received metformin but not quite significantly in either cases (Figure 8B,C, F_(2,19)_ = 4.009, *p* = 0.0922 vs. SC and *p* = 0.0783 vs. HFD, respectively).

No changes in PAC1 and VPAC1 protein expression were observed in the HFD group (*p* > 0.05) and VPAC2 protein expression was not affected either by HFD or metformin administration (Figure 8B,C, *p* > 0.05). However, treatment with metformin evoked a modest downregulation of PAC1 and VPAC1 protein expression, although this was not statistically significant (Figure 8B,C, F_(2,9)_ = 5.044 and F_(2,9)_ = 11.88, *p* = 0.0614 and *p* = 0.0625, respectively).

**Figure 9:**
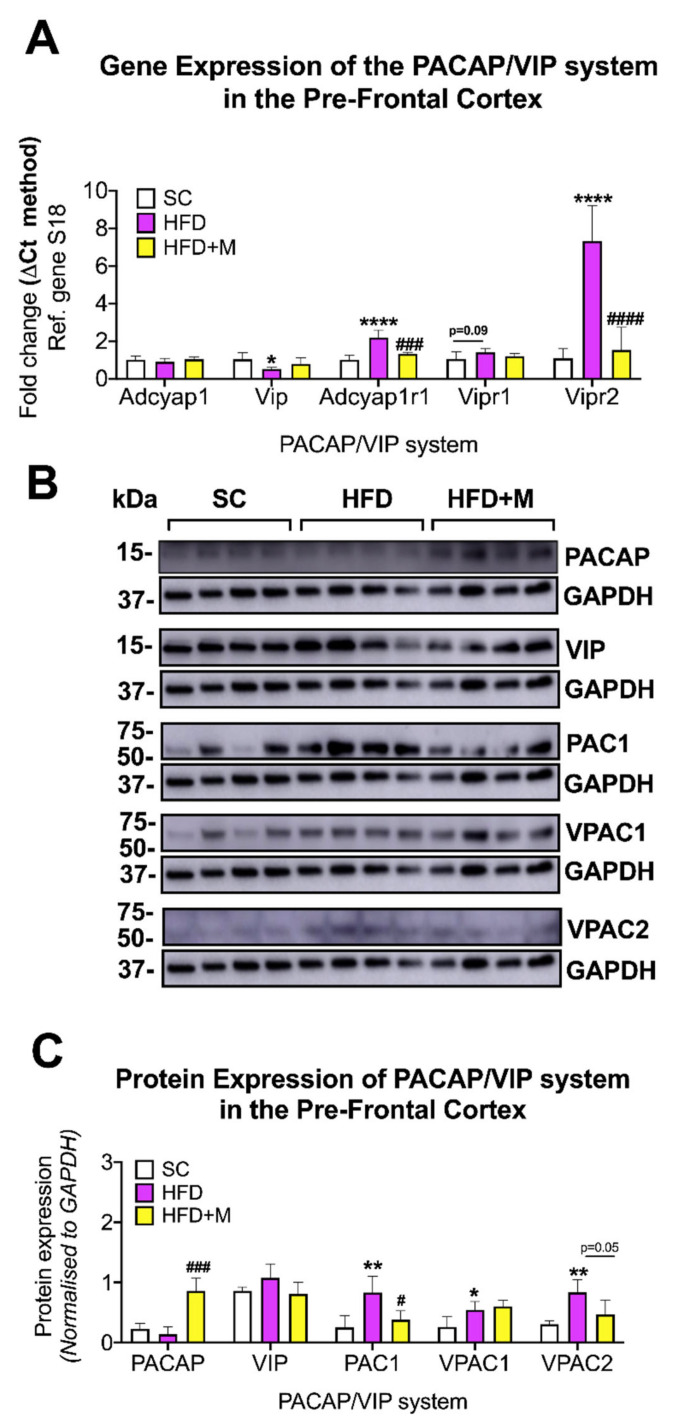
Gene and protein expression analysis of the PACAP/VIP system in the prefrontal cortex of C57BL/6 mice in response to HFD or metformin administration. Mice were grouped as SC, HFD, and HFD + M. Prefrontal cortices were microdissected from the left and right hemibrains from all treatment groups and were used for RNA and protein extraction with subsequent real-time qPCR and Western blot, respectively. (**A**) Differential gene expression levels of the PACAP/VIP system, (**B**) Immunoblot bands for PACAP/VIP system members were represented. (**C**) Protein expression levels of the PACAP/VIP system following normalization to the loading control, GAPDH. mRNA levels were quantified using the ΔCt method and normalized to the reference gene S18 (housekeeping gene). * *p* < 0.05 or **** *p* < 0.0001 vs. SC group, ### *p* < 0.001 or #### *p* < 0.0001 vs. HFD as determined by one-way ANOVA followed by Tukey post-hoc test. *n* = 4–8 per group. Quantification of immunoblot bands was performed using the NIH ImageJ software. Data were given as mean ± S.E.M. * *p* < 0.05 or ** *p* < 0.01 vs. SC group; # *p* < 0.05 or ### *p* < 0.001 vs. HFD group as determined by one-way ANOVA followed by Tukey post-hoc test. *n* = 4 per group.

PFC: At the transcriptional level, *Vip* mRNA was significantly decreased by HFD (Figure 9A, F_(2,15)_ = 3.667, * *p* < 0.05 vs. SC), whereas *Adcyap1r1* and *Vipr2* gene levels showed increases in the HFD group (Figure 9A, F_(2,13)_ = 28.81 and F_(2,11)_ = 30.27, **** *p* < 0.0001 for both genes, respectively). *Vipr1* mRNA levels were also increased in the HFD group; however, the change was not statistically significant (Figure 9A, F_(2,15)_ = 2.592, *p* = 0.0905). Metformin treatment rescued most of the HFD-induced effects, and most notably it reversed *Vip* mRNAs, and decreased *Adcyap1r1* and *Vipr2* transcripts to levels close to those seen in mice fed with a regular diet (SC) (Figure 9A, ### *p* < 0.001 and #### *p* < 0.0001 vs. HFD, respectively). 

Protein expression analyses in the PFC confirmed the stability of PACAP expression in response to the HFD (Figure 9B,C, *p* > 0.05). Nonetheless, we report the unexpected finding of an upregulation of PACAP protein expression in HFD mice that had also received metformin (Figure 9B,C, F_(2,9)_ = 28.21, ### *p* < 0.001 vs. HFD), an effect not seen in VIP expression (*p* > 0.05).

Consistent with mRNA data, HFD increased the expression of PAC1 (F_(2,9)_ = 8.41, ** *p* < 0.01 vs. SC), VPAC1 (F_(2,9)_ = 7.026, * *p* < 0.05), and VPAC2 (F_(2,9)_ = 8.417, ** *p* < 0.01) in the PFC (Figure 9A,B). Metformin treatment reliably prevented PAC1 (# *p* < 0.05 vs. HFD) and VPAC2 increases caused by HFD (*p* = 0.0525 vs. HFD, Figure 9B,C).

**Figure 10:**
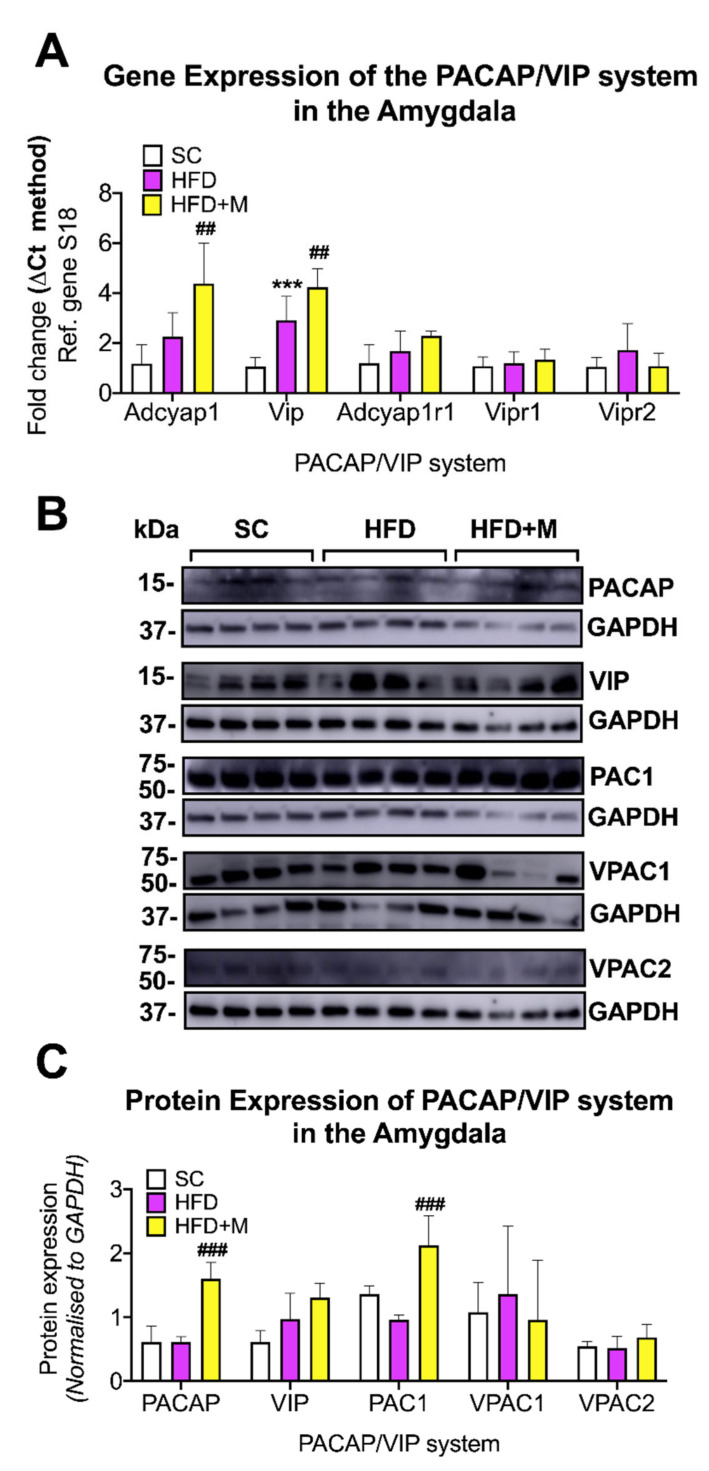
Gene and protein expression analysis of the PACAP/VIP system in the amygdala of C57BL/6 mice in response to HFD or metformin administration. Mice were grouped as SC, HFD, and HFD + M. Amygdala was microdissected from the left and right hemibrains from all treatment groups and used for RNA and protein extraction with subsequent quantitative qPCR and Western blotting, respectively. (**A**) Differential gene expression levels of the PACAP/VIP system, (**B**) Immunoblot bands for PACAP/VIP system members were represented and (**C**) Protein expression levels of the PACAP/VIP system following normalization to the loading control, GAPDH. mRNA levels were quantified using the ΔCt method and normalized to the reference gene S18 (housekeeping gene). *** *p* < 0.001 vs. Std chow; ## *p* < 0.01 vs. HFD group as determined by One-Way ANOVA followed by Tukey post-hoc test. *n* = 6–8 per group. Quantification of immunoblot bands was performed using the NIH ImageJ software. Data were given as mean ± S.E.M. ### *p* < 0.001 vs. HFD group as determined by one-way ANOVA followed by Tukey post-hoc test. *n* = 4 per group.

Amygdala: In the amygdala, dysregulations of the PACAP/VIP system were less pronounced. At the mRNA level, both *Adcyap1* and *Vip* transcripts were upregulated by the HFD; however, only the *Vip* increase was statistically significant (Figure 10A, *** *p* < 0.001 vs. SC). None of the transcripts encoding for the PACAP/VIP receptors were affected by the diet and/or drug treatment (*p* > 0.05).

In the HFD group receiving metformin, *Adcyap1* mRNA was further upregulated (Figure 10A, F_(2,17)_ = 12.21, ## *p* < 0.01 vs. HFD), a finding that was confirmed at the protein level (Figure 10B,C, F_(2,9)_ = 29.17, ### *p* < 0.001 vs. HFD). *Vip* mRNA expression was also further increased after metformin administration to HFD animals (F_(2,18)_ = 28.26, ## *p* < 0.01 vs. HFD); however, protein expression analyses only showed a minor increase in VIP protein expression (Figure 10A–C, *p* > 0.05). 

VPAC1 and VPAC2 protein expression was unchanged in the HFD and HFD + M groups (Figure 10B,C, *p* > 0.05 for both). However, PAC1 protein levels, which were unchanged in the HFD group (*p* > 0.05 vs. SC), were significantly upregulated by metformin treatment (F_(2,9)_ = 18.11, ### *p* < 0.001). 

## 3. Discussion

In the present study, we provide evidence that HFD, in addition to causing metabolic disruptions and weight gain, also triggers the onset of a regionally specific low-to-moderate degree of neuroinflammation and dysregulation of the neuroprotective PACAP/VIP system. These effects were detected in the hypothalamus, hippocampus, and prefrontal cortex, areas with known contributions to the regulation of stress, memory, and decision-making. We also found that neuroinflammation is accompanied by diminished activation of the PI3K/AKT intracellular pathway in the hypothalamus and hippocampus and, to a lesser extent, in the prefrontal cortex. Surprisingly, most of these HFD-induced detrimental effects spared the amygdala, a key brain structure controlling emotions and fear responses. 

We also convincingly demonstrated that metformin, an FDA-approved hypoglycemic drug, can reduce the inflammatory burden caused by HFD. Finally, we report that metformin’s beneficial effects are linked with a robust activation of the PI3K/AKT cascade and the recovery and restoration of the neuroprotective PACAP/VIP system. 

To our knowledge, this is the first study in mice to identify proinflammatory effects of HFD in extra-hypothalamic regions and to validate the efficacy of the oral hypoglycemic agent ‘metformin’ in rescuing neuroinflammation and the local neurochemical alterations triggered by HFD, possibly via a mechanism that involves the activation of the PI3K/AKT pathway.

### 3.1. Establishing a Suitable Animal Model of HFD-Induced Obesity and Assessing the Hypoglycemic Effects of Metformin

In vivo biochemical assessments were conducted to confirm the establishment of a suitable model of HFD-induced obesity, which was able to recapitulate some of the clinical features seen in obese individuals. A previous study has shown that HFD can lead to obesity, hyperinsulinemia, and altered glucose homeostasis due to insufficient compensation by the islets [18], as well as the development of IR [67]. Fasting blood glucose and blood insulin levels demonstrated that mice fed an HFD developed IR, indicated by persistently elevated blood glucose levels identified using two biochemical assays, when compared to mice on a standard diet. These results confirm that prolonged exposure (16 weeks) to a diet rich in fats significantly altered glucose metabolism, caused obvious weight gain, and increased tolerance to insulin (Figure 1A–C). Additionally, HOMA-IR calculations revealed a more than 4-fold increase in IR in HFD-treated animals (Figure 1D). By contrast, administration of the antidiabetic drug metformin improved fasting blood and insulin glucose levels (Figure 1A,B), decreased weight gain (Figure 1C), and reduced IR (Figure 1D), consistent with previous evidence from human [67] and animal studies [68].

### 3.2. High-Fat Diet Triggers Distinct and Regional-Specific Neuroinflammatory Changes and Promotes Glial Activation 

#### 3.2.1. Hypothalamus 

Our findings in the hypothalamus align with previous evidence demonstrating the development of mild neuroinflammatory activity with overt gliosis in this brain region [69,70] and the induction of proinflammatory cytokines in the hypothalamus of mice exposed to similar dietary regimes [71,72]. Based on these data, it appears that an unbalanced diet triggers metabolic alterations in neurons that are detected by resident glia, which release proinflammatory cytokines, and recruit immune cells within the CNS [72,73,74]. Over the long term, it is this sustained neuroinflammatory microenvironment that contributes to dysfunctional hypothalamic activity, which manifests clinically in the appearance of sleep-breathing disorders [75], altered hormonal profiles, decreases in *libido* [76], and changes in feeding habits [77,78] as are often seen in chronically obese patients. 

#### 3.2.2. Hippocampus

We observed the characteristic signatures of mild neuroinflammation and glial activity in the hippocampus of mice fed an HFD. Elevated proinflammatory cytokines, as we report here, have been strongly associated with hippocampal-dependent memory impairments [79,80,81]. It is particularly interesting that hippocampal IFN-γ levels were increased by more than 3-fold in our HFD cohort (Figure 3). IFN-γ plays a critical neuroprotective function specifically preventing injury of hippocampal neurons [82]. Although, it is also reported that IFN-γ can impair hippocampal function as its downregulation is associated with increased hippocampal cell density and synaptic plasticity [83]. Our study calls attention to the complexity of the relationship of IFN-γ levels and hippocampal function and supports the theory that elevated IFN-γ expression in the hippocampus of HFD mice represents an attempt by glial cells to regain homeostatic control within the “hostile CNS microenvironment” triggered by the diet. In contrast, the effects seen in response to metformin treatment are likely associated with the recovery of neurons from the dysfunctional neuroplasticity triggered by HFD [84]. Further investigations assessing the kinetics of IFN-γ expression in the hippocampus at different stages of HFD-induced neuroinflammation and drug treatment are warranted to shed more light on the complex and apparently cell-specific response of IFN-γ. 

#### 3.2.3. PFC

Accumulating evidence suggests that a diet rich in fat may impair cognitive functions controlled by the PFC including object recognition, psychomotor efficiency, and attention as well as affecting individual vulnerability to the development of mood disorders [85,86]. In this study, the PFC was the brain region with the most prominent inflammatory profile in response to HFD, although we found evidence for only moderate glial activation (Figure 4). 

The PFC is recognized for being somewhat resistant to inflammation when compared with other regions, such as the hypothalamus or the hippocampus [87]. Nonetheless, our results showed a strong inflammatory profile in the PFC, suggesting that this brain region might be specifically vulnerable to the effects of a chronic HFD regime. 

It has been reported previously that being fed an HFD for 8 weeks caused a significant downregulation of gamma-aminobutyric acid (GABA) in the PFC of rats, and that this effect can be attributed to the key role elicited by GABA in the regulation of food intake and body weight. GABA also exerts anti-inflammatory and immunomodulatory actions through its inhibitory activity on NF-κB [88]; therefore, it is possible that the protracted (16 weeks) HFD regime used in this study was able to dramatically suppress the PFC anti-inflammatory mechanisms, including the anti-inflammatory GABA/NF-κB axis. In other studies, prolonged HFD was shown to induce chronic stress in animals [77], and the PFC is one of the most vulnerable regions to stress [89]. Therefore, it cannot be excluded that HFD-induced stress may act as a compounding factor that contributes to the exacerbated inflammatory burden seen in the PFC. Finally, we highlight that the only cytokine that was significantly downregulated in the PFC in our model was *IFN-γ* (Figure 4) and note that *IFN-γ* expression is downregulated in conditions that cause chronic stress [90].

#### 3.2.4. Amygdala

An obesogenic diet during critical stages of development can lead to impairment of fear learning/extinction processes as well as precipitating mood disorder-related behaviors in rats. These effects are mediated by alterations to normal brain development and most likely by changes to the amygdala. The amygdala is a region sensitive to the central metabolic changes induced by HFD. To our surprise, we did not identify any significant changes in the expression of proinflammatory cytokine mRNAs, nor was there evidence of glial activation in the amygdala of mice fed an HFD (Figure 5). The reasons that the amygdala was unaffected by HFD are not clear. The amygdala comprises five main subnuclei, each of which show differing degrees of sensitivity to stressors and related physiological responses. In this study, our tissue blocks included the entire amygdaloid complex. It is possible that only specific nuclei are vulnerable (or respond) to HFD, for example, the basolateral amygdala has been identified as being vulnerable in previous work [91]. Further investigations addressing the effects of HFD in specific amygdaloid subnuclei will help to better understand the possible heterogeneities of response to dietary stress within the amygdala. 

### 3.3. Metformin Ameliorates HFD-Induced Neuroinflammation 

Metformin is used as a first-line therapy drug for the treatment of T2D [35]. Metformin’s peripheral activity in regulating glucose metabolism and insulin resistance is well understood; however, it is only recently that anti-inflammatory effects on the brain have been identified for this compound [92,93]. In this study, metformin administration ameliorated inflammation triggered by HFD and partially suppressed glial activation in the four brain regions examined (Figure 2, Figure 3, Figure 4 and Figure 5). The elevated mRNA levels of the hypothalamic inflammatory cytokines (i.e., *IL-6* and *Mcp-1*) were downregulated in the cohort of mice that received metformin. Similarly, microglial activity (iNOS) and astrocyte activity (GFAP) were also reduced by the drug (Figure 2), confirming data from previous reports [92]. Similar effects of metformin were seen in both the hippocampus and PFC. However, HFD-induced inflammatory activity differed significantly between these two brain regions, with a 1.5- to 3-fold upregulation of proinflammatory cytokines in the hippocampus, and a 2.0- to 7.0-fold increase in the PFC. The reasons behind the differing responses to the same metabolic insult in the two regions are unclear, but as discussed above, it cannot be excluded that other compounding factors (i.e., stress triggered by the diet) play a significant role. Notwithstanding these differences, metformin treatment abrogated inflammation in both brain regions irrespective of the neuroinflammatory burden. Notably, in the PFC, metformin did not reverse the HFD-evoked downregulation of *IFN-γ* mRNA (Figure 4). As discussed above, it is possible that *IFN-γ* transcriptional activity is reduced due to increased stress triggered by HFD via a mechanism that metformin is unable to affect. 

The HFD did not affect either the inflammatory profile or levels of glial activity of the amygdala. The expression of proinflammatory cytokines, microglia, or astrocyte activation markers was also unaffected in HFD animals that received metformin with the exception of *Mcp1* mRNAs, which were significantly downregulated by metformin administration (Figure 5). Interestingly, our results indicate that metformin’s activity does not interfere with regional brain homeostasis in the absence of overt signs of inflammation, as seen as in the amygdala. This point is of relevance when considering future clinical applications of metformin in the treatment of dietary-induced CNS conditions, as the drug seems to specifically target the inflamed CNS, sparing healthy/unaffected brain structures.

### 3.4. Metformin Activates the PI3K/AKT Pathway in the Brain of HFD Mice 

There is evidence supporting a role for metformin in the improvement of cognitive function in patients with abnormal glucose metabolism [94]. It evokes anti-inflammatory activity and resets metabolic activity in the brain [94,95]. It is suggested that the anti-inflammatory activities of metformin in the brain are mainly through its activity on AMPK [38,94]. In this study, we tested if the downstream target of AMPK, the PI3K/AKT pathway, was affected in response to HFD or metformin treatment. Intriguingly, our first finding showed that the AKT phosphorylation at ser-473 residue was reduced following HFD and it was fully rescued following metformin administration in both the hypothalamus, hippocampus, and PFC (Figure 6A–C). This pathway is well-known to reduce proinflammatory processes [96,97,98]; therefore, our data corroborates the idea that metformin treatment may attenuate inflammation via this intracellular pathway. 

AKT phosphorylation in the amygdala showed no evidence of PI3K/AKT activation in HFD mice (Figure 6D). This could be due to the low/absent inflammation observed in this brain region (Figure 5). However, metformin treatment robustly upregulated p-AKT activity, suggesting that the drug activates this intracellular pathway independently of metabolic damage, such as that caused HFD in other brain regions. 

### 3.5. PACAP/VIP System in Response to Chronic HFD and Metformin Administration 

The PACAP/VIP system is known for its powerful immune modulatory and neuroprotective actions, mediated through the receptors PAC1, VPAC1, and VPAC2r [44]. The effects of HFD and metformin administration on the PACAP/VIP system were evaluated.

#### 3.5.1. Hypothalamus 

In the hypothalamus, PACAP and VIP are well known to regulate both appetite and energy homeostasis [50], as well as feeding behaviors and circadian rhythm [51]. Our data showed that PACAP and VIP expression levels were reduced in mice fed HFD (Figure 7A–C). This was unexpected, especially given the anti-inflammatory activities of both peptides in response to inflammation [44]. However, it should be noted that PACAP expression is mostly restricted to the median eminence [99], a BBB-free area of the hypothalamus regarded as “structurally weak” [100]. The anatomical vulnerability combined with the negative impacts of inflammation on the BBB may have contributed to dampening the endogenous anti-inflammatory mechanisms protecting the CNS, and thereby causing the PACAP downregulation we observed in the hypothalamus. 

Unlike PACAP, VIP is found in high concentrations in both the suprachiasmatic nucleus [101] and the paraventricular nucleus of the hypothalamus [102], where it plays an integral role in regulating circadian rhythms and prolactin secretion [103]. Interestingly, VIP in neurons of the paraventricular nucleus regulate prolactin secretion through their projections to the median eminence [102], thus it is possible that VIP downregulation in response to HFD could be indirectly caused by its neuronal connections to the median eminence.

In contrast to the general downregulation of PACAP and VIP that we observed, there was a marked upregulation in PAC1 protein and VPAC2r mRNA levels in response to HFD (Figure 7A–C). Animals fed an HFD develop stress and manifest anxiety-like behaviors, possibly as a direct consequence of the dietary change [77]. PAC1 is critically involved in modulating stress responses through the HPA axis, and PAC1 levels are upregulated in response to chronic stress [104,105]. Moreover, hypothalamic PAC1 and VPAC2r mediate anorexigenic effects [106,107], which could also explain the upregulation seen in HFD mice, as this could reflect a response to increased fat intake and the associated stress. 

Consistent with the ability of metformin to alleviate hypothalamic inflammation, we report that the drug also rescued all the alterations of the PACAP/VIP system caused by the HFD (Figure 7A–C). 

#### 3.5.2. Hippocampus 

In the hippocampus, the PACAP/VIP system was not affected by HFD. In fact, although PACAP, PAC1, and VPAC2 mRNA expression levels were upregulated, there were no changes in their protein expression levels in HFD mice (Figure 8A–C). With the knowledge that the hippocampus is one of the vulnerable regions of the CNS, it is possible that this neuroprotective system is endogenously activated in response to HFD feeding. Although the mRNA inflammatory profile expression indicated the presence of inflammation in this region, this trend was not reflected at the protein level. The failure to detect significant inflammation in the hippocampus in response to HFD may be due to increased anti-inflammatory activity of PACAP through its receptors. PACAP/VIP receptors are also expressed in microglia [44], and there is strong evidence that PACAP can modulate microglial response and inhibit the release of inflammatory mediators [108]. The proinflammatory markers *IL-1α*, *IL-6*, *TNF*, and *Mcp1* were not elevated in response to HFD in the hippocampus (Figure 3) whereas in other ROIs (i.e., PFC and hypothalamus) where the PACAP/VIP system was dysregulated, these proinflammatory markers were significantly upregulated. The increase in PACAP and PAC1 in the hippocampus seems to be important for maintaining and enhancing learning and memory functions [109]. Our data provides further indications regarding the role of this protective system, which could aid in our understanding of the negative impacts of HFD on hippocampal-mediated learning and memory functions. 

In contrast to PACAP, regulation of hippocampal VIP expression was decreased in HFD animals (Figure 8B,C). This could be due to the natural co-localization of VIP expression on GABA-ergic neurons of the hippocampus [110]. GABA-ergic neurons control neuronal excitability, the processing of information, plasticity, and the synchronization of neuronal activity [111]. Interestingly, hippocampal GABA-ergic neurons are negatively impacted by HFD feeding in rats [112], thus the depressed expression of VIP on these neurons could explain our findings. 

As for the other brain regions, metformin treatment recovered the levels of the hippocampal PACAP/VIP system members that were disturbed by HFD back to control levels (Figure 8A–C).

#### 3.5.3. PFC

Our major finding in the PFC is the downregulation of VIP and the upregulation of the receptors PAC1 and VPAC2 with a slight increase in VPAC1 mRNA levels (Figure 9A). 

The dysregulation highlighted above seems to be related to the inflammation caused by HFD in the PFC. These PACAP/VIP alterations could exacerbate the negative impacts of the HFD by increasing stress behaviors. These findings highlight the sensitivity of the PFC and of the PACAP/VIP system to different dietary regimes and reveal another factor that may contribute to the development of cognitive impairments in chronically obese patients [113]. However, it is imperative to test other pathways, such as the HPA axis, which is also partly regulated by the PFC [105].

In the PFC, metformin treatment restored the altered levels of PACAP/VIP peptides as well as receptors to levels comparable to controls (Figure 9A–C) as it did in the other regions evaluated. In addition, it strongly increased PACAP protein expression, further supporting the neuroprotective effects of metformin on this system.

#### 3.5.4. Amygdala

PACAP and VIP in the amygdala are involved in the modulation of stress and fear responses [114] as well as the emotional regulation of feeding behavior (i.e., satiety) [50] via connections with the hypothalamus and other brain regions [115]. Our results indicated increased VIP transcripts in the amygdala of mice fed an HFD (Figure 10A). VIP exerts potent anorexigenic effects [116,117], thus it is possible that this increase could be a physiological negative feedback response to induce the sense of satiety and inhibit food craving in animals subjected to the dietary regime. 

PACAP peptide, as well as PAC1, VPAC1, and VPAC2 receptors, were unaffected by HFD (Figure 10A–C). Given the absence of inflammation in this brain region (Figure 5), the stability of PACAP and receptors under these experimental conditions is perhaps not surprising [118]. 

Relatively little is known about metformin’s effects in the amygdala [119]. HFD-fed mice that were co-treated with metformin showed an interesting pattern of upregulation of both PACAP and VIP at the mRNA level, with the increase in PACAP also confirmed at the protein level (Figure 10A–C). To our knowledge, there are no studies providing any direct links between the PACAP/VIP system and metformin. However, the upregulation of these peptides by metformin treatment in the absence of HFD-induced inflammation could be due to the development of insulin resistance (IR) in this region. Studies have shown that during HFD, signs of brain IR can develop prior to overt neuroinflammation [120]. Since the PACAP/VIP system has been associated with modulating IR in the hyperinsulinemia environment, possibly by sensitizing neurons to insulin [121], it is possible that metformin increased the expression of PACAP and VIP in an attempt to regain homeostatic control of brain glucose metabolism, noting that metformin is known to sensitize neuronal cell lines to insulin under hyperinsulinemic insults [119]. 

## 4. Materials and Methods

### 4.1. Animals 

Five-week-old male C57BL/6 mice were acquired from Australian BioResources (Moss Vale, NSW, Australia). The mice were housed in standard cages with a maximum of five mice per cage. The animal housing conditions were maintained on a 12 h light/dark cycle at a room temperature of 22.5 ± 2 °C. 

**Figure 11:**
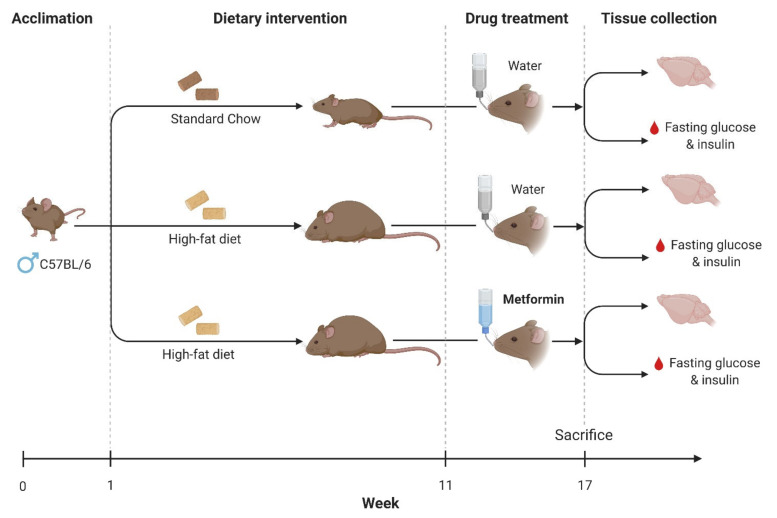
Experimental design. Schematic diagram depicting the experimental design consisting of a dietary intervention to induce obesity and insulin resistance (high-fat diet) and pharmacological treatment (metformin) to ameliorate brain inflammation. At the end of the experimental protocol, brains and blood samples were collected for downstream analyses.

Mice were allowed 1 week to acclimate to housing conditions, during which time they were provided *ad libitum* access to standard laboratory chow and tap water. After acclimatization, animals were ear-notched and either remained on a standard chow (SC) (6% fat, Gordon’s Specialty Stock Feeds) or were shifted to a high-fat diet regime (HFD) (22% fat Glen Forest Specialty Foods) for a period of 16 weeks. After 11 weeks of the HFD regime, half of the animals on an HFD were administered metformin via their drinking water at a concentration of 30 mg/mL. Control mice received plain drinking water only. Thereafter, at week 17, all animals were euthanized (please refer to Figure 11 below). Efforts were made to minimize animal suffering and to reduce the number of animals used. All experiments were conducted in line with the “Australian Code of Practice for the Care and Use of Animals for Scientific Purposes”. All procedures were approved by the University of Technology Sydney Animal Care and Ethics Committee (Animal Ethics approval: *UTS ACEC 2015000684*, approved on 12 May 2015). 

### 4.2. Drug Administration 

Metformin was administered via drinking water at a concentration of 30 mg/mL. Control mice received plain drinking water only. Metformin treatment commenced after the mice were given HFD for 11 weeks and continued until animals were sacrificed in week 17 (total treatment duration = 5 weeks). HFD duration and metformin dose regimes were based on previous studies demonstrating significant increases in blood glucose levels and insulin resistance after 11 weeks on an HFD regime and beneficial effects of metformin on several biochemical parameters at the given dose [122,123,124,125,126,127]. 

### 4.3. Fasting Glucose and Insulin Measurements

At the end of the study, mice were fasted for 5 h before blood glucose levels were measured using a glucometer (Accu-Check Performa, Roche Diagnostics, North Ryde, NSW, Australia). Plasma insulin levels were measured using a mouse BioPlex kit (Bio-Rad, Hercules, CA, USA). The homeostatic model assessment of insulin resistance (HOMA-IR) was determined using the following equation: HOMA-IR = fasting glucose (mg/dL) × fasting insulin (μU/mL)/405, as previously reported [128]. During the in vivo experiments, the weights of the animals were monitored on a weekly basis and measurements were recorded. 

### 4.4. Method of Euthanasia

At week 17, immediately after biochemical assessments, animals were euthanized humanely. Mice were anaesthetized with isoflurane in an induction chamber until no reflex to pedal or ocular stimulation was observed. To ensure that the mouse was deceased before any further procedures, rapid cervical dislocation was performed, and then the head was removed and the brain collected.

### 4.5. Tissue Collection and Microdissections 

The brains were placed in liquid nitrogen, and then stored at −80°C. For micro-dissections, brains were placed on an ice-cold metal tray and selected blocks containing the brain regions of interest (ROI) from each side (left and right hemispheres) were cut using a razor blade pre-treated with RNAlater (Sigma-Aldrich, St. Louis, MO, USA) under a stereoscopic microscope equipped with an 8×-objective. A mouse brain atlas was used as a reference [129]. Tissue blocks consisted of the prefrontal cortex (PFC), hypothalamus, hippocampus, and amygdala. Each brain region was weighed using a high-precision scale. After dissection, all specimens were immediately snap frozen in liquid nitrogen in 1.5 mL RNase/DNase-free microcentrifuge tubes, and then stored at −80°C. Blocks from the left hemisphere were utilized for mRNA analyses, whereas the right hemisphere was used for protein expression analyses.

### 4.6. RNA Extraction, cDNA Synthesis, and Real-Time Quantitative Polymerase Chain Reaction

RNA was extracted from the left prefrontal cortex, left half of the hypothalamus, left hippocampus, and left amygdala of the SC, HFD, and HFD + M mice group. RNA was isolated using 1 mL of TriSure reagent (Bioline, Eveleigh, Australia) and 0.2 mL of chloroform and precipitated with 0.5 ml of isopropanol. Pellet was washed in 1 mL of 75% ethanol and air dried. cDNAs were synthesized using 1 µg of RNA from each sample diluted with milliQ H_2_O in a final volume of 15 μL. In total, 5 μL of cDNA synthesis mix (SensiFast cDNA synthesis kit (Bioline, Redfern, Australia), consisting of (per sample) 4 μL of TransAmp Buffer and 1 μL of reverse transcriptase were added to each diluted RNA sample (final volume = 20 μL). Mixtures were then incubated at 45 °C for 40 min to initiate the synthesis process. The reaction was terminated by incubation of samples at 85 °C for 5 min. Aliquots of cDNA from all ROI of SC, HFD, and HFD + M were amplified in parallel reactions, using the primers indicated in Table 1. The S18 ribosomal protein subunit was used as the housekeeping gene in all qPCR reactions. Each reaction was made up to a final volume of 10 μL for use in the BIO-RAD CFX96 Real-Time instrument. Each reaction included 3 μL of diluted cDNA (final concentration 30 ng/ µL), 5 μL of SensiFAST SYBR^®^ No-ROX master mix (Bioline), 0.8 μL of 5 μM forward primer, 0.8 μL of 5 μM reverse primer, and 0.4 μL of MilliQ H_2_O. The amplifications were performed using the BIO-RAD CFX96 Real-Time instrument with the following program setting: (I) initial denaturation (1 cycle: 95 °C for 2 min); (II) denaturation, annealing, extension (40 cycles: 95 °C for 5 s, 60–65 °C for 10 s, 72 °C for 5–20 s). PCR products’ specificity was evaluated by melting curve analysis. 

To investigate the different expression levels, we analyzed the mean fold change values of each sample calculated using the ΔΔCt method [130]. For the quantification of each gene, we considered the SC group as the internal calibrator. The ΔΔCt of each sample was then calculated by subtracting the calibrator ΔCt to the sample ΔCt. The formula 2^−ΔΔCt^ was used to calculate fold changes. Baseline measurements for each calibrator sample were set to 1.

### 4.7. BCA Protein Quantification Assay

In order to quantify the protein concentration of each tissue sample, we used the Bicinchoninic-Acid Assay (BCA) kit from Thermo Scientific. Bovine-Serum-Albumin (BSA) was used to generate a standard curve. Increasing BSA amounts with known concentrations along with the unknown protein samples were loaded into 96-well plates. After that, the working reagent was prepared by mixing 50 parts of Reagent A with 1 part of Reagent B. The mixture was then vortexed and loaded into the wells. In total, 200 μL of working reagent were required for each well containing the unknown protein or the standards. The plate was then incubated at 37 °C for 40 min. Thereafter, the plate was cooled at room temperature for 10 min and measurements were then taken using the TECAN-infinite M1000-PRO ELISA reader. Optical densities were measurements at absorbance set at 562 nm. 

### 4.8. Western Blot Analyses 

Sample proteins (30 μg) were diluted in 2× Laemmli buffer (Invitrogen, Carlsbad, CA, USA), heated at 70 °C for 10 min, separated on a Biorad Criterion XT 4–20% gradient Tris-glycine polyacrylamide gels (Invitrogen) by electrophoresis, and then transferred to (Polyvinylidene difluoride) PVDF membranes using the semi-dry approach (Biorad Trans-Blot^®^ Turbo Transfer System). Briefly, the gel was assembled in a sandwich with filtering papers and a PVDF membrane and then placed into a cassette and transferred for 7 min using a pre-set voltage (12.5 mA, 30 V). After being blocked for 1 h in 5% skim milk in TBST at room temperature, membranes were incubated overnight at 4 °C with different primary antibodies (please refer to Table 2) in 1% skim milk containing 1X TBST. Membranes were then washed and incubated with secondary antibody raised against the primary animal in 5% skim milk and TBST 1X for 1 h. The densities of the bands on the membrane were scanned and analyzed using the chemiluminescent approach on an Amersham Imager 600 System.

### 4.9. Statistical Analyses 

Statistical analyses were computed using GraphPad Prism software ver. 7.02, GraphPad Software, San Diego, CA, USA, www.graphpad.com (accessed on 19 August 2018). All values are reported as means ± SEM. All the analyses were carried using One-Way Analysis of Variance (ANOVA), followed by Tukey post-hoc analyses to assess for significance among groups. *p*-values less than 0.05 denoted statistical significance.

## 5. Conclusions

In summary, this study showed that HFD causes sustained and low/moderate CNS inflammation that, in addition to the hypothalamus, also affects extra-hypothalamic structures, such as the PFC and the hippocampus. In addition, our findings indicate that the inflammatory profile is distinct for each examined brain region, supporting the notion that CNS responses to HFD are region specific and may involve the activation of multiple adaptive mechanisms. In fact, we cannot exclude that the inflammatory *milieu* seen in each CNS region after HFD might be the cumulative outcomes from heightened inflammation and defective responses of the protective PI3K/AKT pathways and the PACAP/VIP system. The results obtained with metformin support this theory, as the drug restores both protective pathways and attenuates inflammation. Although it is critical to fully characterize the full spectrum of positive effects (and adverse effects) of the drug in the CNS, the results pinpoint the usefulness of repurposing metformin as a therapeutic option to treat CNS disorders characterized by the presence of an underlying neuroinflammatory component, such as multiple sclerosis or other neurodegenerative diseases.

## Figures and Tables

**Table 1:** Primer sequences targeting the PACAP/VIP system and the inflammatory markers optimized for real-time quantitative PCR. Predicted amplicon lengths are indicated in base pairs (bp).

Gene (Ref. Seq.)	Primers	Location of Primers	Tm (°C)	Length (bp)
PACAP peptide (NM_009625.2)	5′-CTGCGTGACGCTTACGCCCT-3′ 3′-CCTAGGTTCTCCCCCGCGCC-5′	401 552	59.77 61.02	152
VIP peptide (NM_011702.2)	5′-TGGCAAACGAATCAGCAGCAGCA-3′ 3′-AGCCATTTGCTTTCTGAGGCGGG-5′	487 592	60.18 59.99	106
PAC1 receptor (NM_007407.3)	5′-CAGTCCCCAGACATGGGAGGCA-3′ 3′-AGCGGGCCAGCCGTAGAGTA-5′	1474 1612	59.92 59.40	139
VPAC1 receptor (NM_011703.4)	5′-TCAATGGCGAGGTGCAGGCAG-3′ 3′-TGTGTGCTGCACGAGACGCC-5′	1311 1437	59.71 60.25	127
VPAC2 receptor (NM_009511.2)	5′-GCGTCGGTGGTGCTGACCTG-3′ 3′-ACACCGCTGCAGGCTCTCTGAT-5′	127 281	60.32 60.24	155
Interleukin 1-α (NM_010554.4)	5′-ACGTCAAGCAACGGGAAGAT-3′ 3′- AAGGTGCTGATCTGGGTTGG-5′	229 352	59.97 59.96	124
Interleukin 1-β (NM_008361.4)	5′-GCTACCTGTGTCTTTCCCGT-3′ 3′- CATCTCGGAGCCTGTAGTGC-5′	293 456	59.68 60.25	164
Interleukin 6 (NM_031168.2)	5′-CCCCAATTTCCAATGCTCTCC-3′ 3′-CGCACTAGGTTTGCCGAGTA-5′	578 718	59.24 60.11	141
Interleukin 10 (NM_010548.2)	5′-GCATGGCCCAGAAATCAAGG-3′ 3′-GAGAAATCGATGACAGCGCC-5′	367 457	59.54 59.42	91
Interferon-γ (NM_008337.4)	5′-AGCAAGGCGAAAAAGGATGC-3′ 3′-TCATTGAATGCTTGGCGCTG-5′	425 507	59.76 59.83	83
Tumor necrosis factor (NM_013693.3)	5′-ATGGCCTCCCTCTCATCAGT-3′ 3′-TTTGCTACGACGTGGGCTAC-5′	364 460	60.03 60.39	97
Monocyte chemoattractant protein 1 (NM_011333)	5′-TGACCCCAAGAAGGAATGGG-3′ 3′-ACCTTAGGGCAGATGCAGTT-5′	313 416	59.30 59.00	104
Ribosomal protein S18 (NM_011296.2)	5′-CCCTGAGAAGTTCCAGCACA-3′ 3′-GGTGAGGTCGATGTCTGCTT-5′	36 180	59.60 59.75	145
Glial Fibrillary Acidic Protein (NM_001131020.1)	5’-GCGAAGAAAACCGCATCACC-3′ 3′-TCTGGTGAGCCTGTATTGGGA-5′	1189 1338	60 61	150
Ionized calcium-Binding Adapter molecule 1 (NM_001361501.1)	5′-GCTTTTGGACTGCTGAAGGC-3′ 3′- GTTTGGACGGCAGATCCTCA-5′	402 515	60.04 61.45	114
Cluster of Differentiation 68 (NM_001291058.1)	5′-CTCCCACCACAAATGGCACT-3′ 3′-CTTGGACCTTGGACTAGGCG-5′	386 480	60.54 60.11	95

**Table 2:** Primary antibodies used for Western blotting. Optimal dilutions are shown in the right column.

Antibody Name	Cat. #	Molecular Weight (kDa)	Dilution
Pituitary Adenylate Cyclase-Activating Peptide (PACAP)	GTX37576	18	1/500
Vasoactive Intestinal Polypeptide (VIP)	GTX129461	18	1/300
Pituitary Adenylate Cyclase 1 Receptor (PAC1)	GTX30026	53	1/1000
Vasoactive Intestinal Polypeptide Type-1 Receptor (VPAC1)	LS-C177415	49	1/1000
Vasoactive Intestinal Polypeptide Type-2 Receptor (VPAC2)	ab28624	50–60	1/1000
Glial Fibrillary Acidic Protein (Iba1)	GTX100042	15	1/500
inducible Nitric Oxide Synthase (iNOS)	GTX60599	32	1/500
Glial Fibrillary Acidic Protein (GFAP)	G4546	50	1/500
Phosphorylated protein kinase B (p-Akt)	4060 Ser473	60	1/1000
Protein Kinase B (Akt)	4691 C67E7	60	1/1000
Glyceraldehyde 3-phosphate dehydrogenase (GAPDH)	VPA00187	37	1/1500

## Data Availability

Data is contained within the article or Supplementary Materials.

## Supplementary Materials

The following are available online at https://www.mdpi.com/article/10.3390/ijms222413660/s1.
