# Supplementary figures and images for: Metformin Treatment Attenuates Brain Inflammation and Rescues PACAP/VIP Neuropeptide Alterations in Mice Fed a High-Fat Diet
Source: Int J Mol Sci. 2021 Dec 20;22(24):13660. doi: 10.3390/ijms222413660 (PMC8706124; doi:10.3390/ijms222413660)

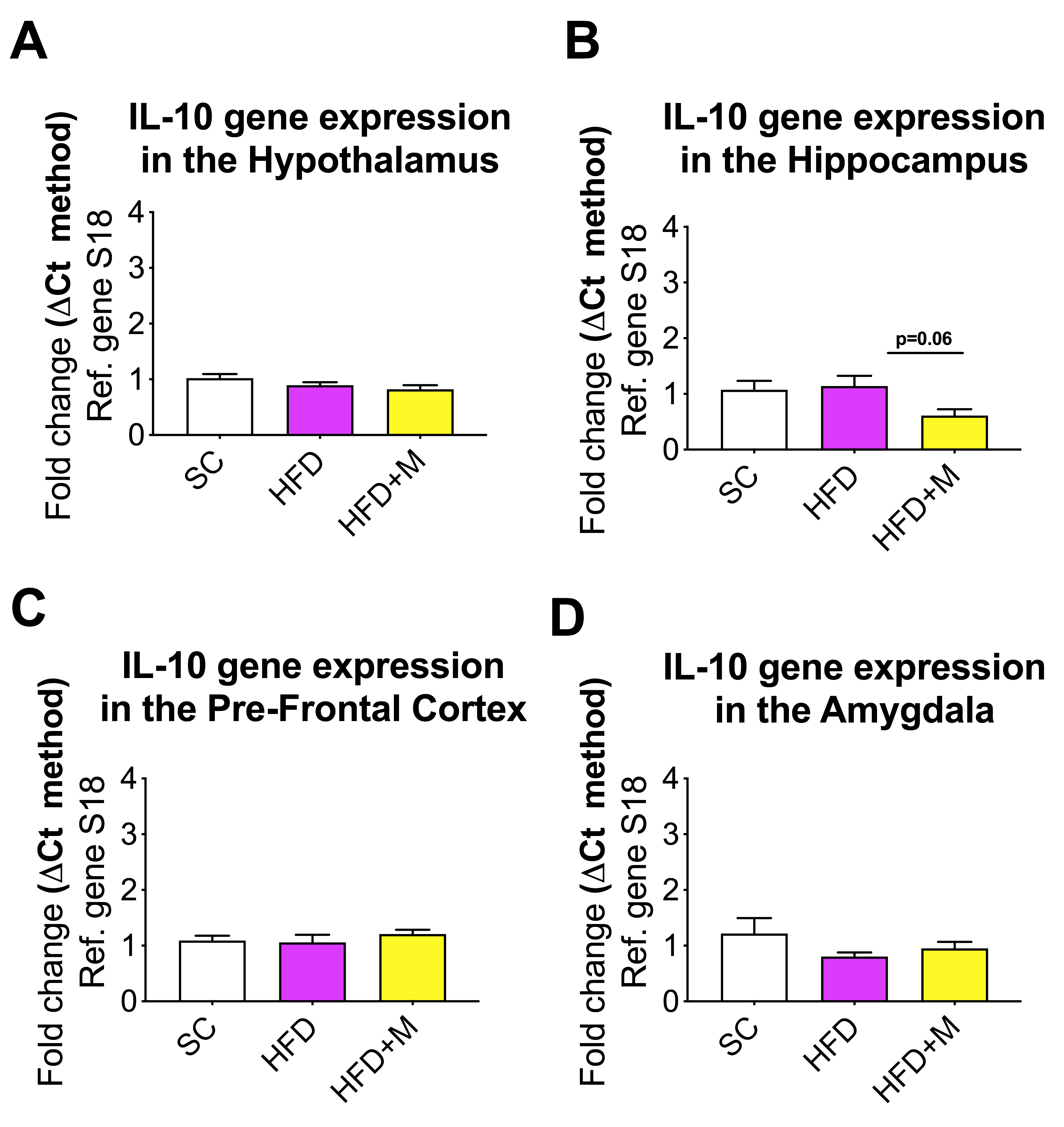

Supplement: Supplementary file 1 [file ijms-22-13660-s001.zip › ijms-1510037-supplementary.tiff]
